# D-mannose promotes the degradation of IDH2 through upregulation of RNF185 and suppresses breast cancer

**DOI:** 10.1186/s12986-023-00774-9

**Published:** 2024-01-02

**Authors:** Ruonan Zhang, Ziyin Tian, Yanping Xu, Lei Lv

**Affiliations:** Nourse Centre for Pet Nutrition, 241200 Wuhu, China

**Keywords:** D-mannose, IDH2, RNF185, NADPH, Breast cancer

## Abstract

**Background:**

D-mannose, an epimer of glucose, which is abundant in some fruits, such as cranberry, has been previously reported to inhibit urinary tract infection. In recent years, the potential function of D-mannose has been broadened into the regulation of other inflammation diseases and cancer. It was reported that D-mannose can increase reactive oxygen species (ROS) production, while IDH2 is important for the generation of NADPH, the crucial reducing factor. These findings prompted us to determine whether D-mannose can regulate IDH2 and IDH2-mediated NADPH production in tumor.

**Methods:**

The breast cancer cell line MDA-MB-231 was cultured and treated with 100mM D-mannose. IDH2 expression was detected by Western Blot and qRT-PCR. RNA-seq was conducted to identify the differentially expressed genes. BioGRID database was used to find the IDH2 interactors. Tumor cells were collected to measure the NADPH production using the NADP+/NADPH detection Kit. Colony formation assay and CCK-8 assay were conducted to evaluate the proliferation of cells.

**Results:**

D-mannose can promote IDH2 protein degradation through ubiquitination-proteasome pathway. Mechanistically, D-mannose treatment upregulated the expression of an E3 ligase - RNF185, which can interact with IDH2 and promotes its proteasomal degradation. Consequently, IDH2-mediated NADPH production was inhibited by D-mannose, the proliferation of breast cancer cells was retarded, and the sensitivity to pro-oxidant of breast cancer cells was elevated.

**Conclusions:**

Our study demonstrated that D-mannose can degrade IDH2 and inhibit the production of NADPH to suppress the proliferation of breast cancer cells and render the breast cancer cells more sensitive to pro-oxidant treatment. Furthermore, we illustrated the E3 ligase RNF185 plays an important role in D-mannose-mediated proteasomal degradation of IDH2.

## Background

Breast cancer is estimated to be the second most common cancer, which accounts for around 11.9% of all cancer types, and ranks 5th as a cause of human death (6.4%) [1]. In women, breast cancer is the most frequently diagnosed cancer and ranks second among causes of cancer-related death [2]. Currently, various therapies are used to treat breast cancer, such as hormone therapy, chemotherapy, immunotherapy, and targeted therapy, as well as the combination of two or three well-established therapies [3]. However, resistance to current therapies still exist, further investigation into the molecular mechanisms underlying tumorigenesis and progression will shed light on the development of new therapeutic approaches in breast cancer treatment.

Isocitrate dehydrogenase 1 and 2 (IDH1 and IDH2) are two key metabolic enzymes, converting isocitrate to α-ketoglutarate (α-KG), utilizing NADP + as a cofactor and producing one equivalent of NADPH. IDH1 is located in the cytosol and peroxisome, while IDH2 is located in mitochondria [4]. IDH1/2 represents one of the most important pathways for the generation of NADPH, an essential reducing factor that controls cellular defense mechanisms against oxidative damage through the reduction of glutathione and thioredoxins [5]. IDH1 and IDH2 are frequently mutated in various types of cancer, including glioma, acute myeloid leukemia, cartilaginous tumors, etc. [6]. IDH1/2 mutations result in simultaneous loss of their normal catalytic activity, the production of α-KG, and gain of a new function, the production of a oncometabolite 2-hydroxyglutarate (2-HG) [7]. While most studies focused on the role of IDH1/2 mutation in cancer, several studies have also revealed the oncogenic role of wild-type IDH1/2 in different cancer types in recent years [8, 9]. In breast cancer, IDH2 was reported to contribute to cell proliferation, anchorage-independent growth, glycolysis, mitochondrial respiration, and antioxidant defense [8], and be associated with an aggressive phenotype of breast carcinoma [10]. Furthermore, IDH2 has been evaluated as an independent risk factor for shorter breast cancer specific-survival based on the transcriptomic and proteomic analysis of IDH2 expression in breast cancer tissue microarrays [11]. Therefore, wildtype IDH2 is a potential target for clinic treatment of breast cancer, and further studies about the regulation of IDH2 and drugs targeting this regulation are warranted.

D-mannose, a C-2 epimer of glucose, is extensively studied in recent years for its function in regulating various pathways in immune cells and tumor cells [12–16]. Our previous study found that D-mannose can promote the degradation of PD-L1 protein, which inspired us to discuss the possibility of D-mannose’s impact on the stability of other proteins [16]. In this study, we found that D-mannose treatment can promote the degradation of IDH2 protein in mammary cancer cells by upregulating the E3 ligase RNF185, rendering the tumor cells more vulnerable to pro-oxidant treatment and inhibiting the proliferation of breast cancer cells.

## Methods

### Cell culture and transfection

Human breast cancer cell line MDA-MB-231, canine breast cancer cells, and 293T cells were cultured in Dulbecco’s modified Eagle’s medium (DMEM) supplemented with 10% fetal bovine serum (FBS) and 1% penicillin/streptomycin (P/S). Cells were maintained at 37 °C and 5% CO2 in a humidified environment. For plasmid transfection, cells were transfected with plasmids using EZ-trans reagent (Life-iLab, Shanghai). For siRNA transfection, Lipofectamine RNAiMAX (Thermo Fisher) was used according to the manufacture’s protocol.

### Quantitative real-time PCR analysis

Total RNA was extracted using EZ-press RNA Purification Kit (EZBioscience) and then subjected to cDNA synthesis using the 4×Reverse Transcription Master Mix (EZBioscience). The expression of targeted genes was detected by quantitative real-time PCR (qRT-PCR) using the qPCR SYBR Green Master Mix (Yeasen, Shanghai). All experimental procedures were performed according to the manufacture’s protocols. The Ct values were analyzed using the 2^−ΔΔCt^ method and the final results were normalized to the expression level of Actin, which served as an internal control. The sequences of primers (synthesized by BioSune, Shanghai) used for qRT-PCR are shown in Table 1.

**Table 1:** Primers used for qRT-PCR analysis

Primers	Forward 5’-3	Reverse 5’-3
Actin	GGCATAGAGGTCTTTACGGATGTC	TATTGGCAACGAGCGGTTCC
IDH2 RNF185	CGCCACTATGCCGACAAAAG GTGTTTACATCAGTGGTTGGAGA	ACTGCCAGATAATACGGGTCA GTGCTGCCCCTTCCATAGAG

### Western blot and co-immunoprecipitation

After treatment, cells were harvested and lysed in NP-40 lysis buffer containing 1% protease inhibitor and 1% phosphatase inhibitor at 4 °C for 30 min. Then the cell lysates were mixed with SDS-PAGE sample loading buffer and heated at 95 °C for 15 min. For co-immunoprecipitation, the cell lysates were centrifuged (12,000 rpm, 15 min, 4 °C) to get the supernatants. Then the supernatants were incubated with anti-FLAG beads (AbHO, Shanghai) overnight at 4 °C. After incubation, the beads were washed 3 times with NP-40 buffer and heated with SDS-PAGE sample loading buffer. After the sample preparation performed as above, western blot was performed to analyze the expression level of target proteins according to the standard protocol. Antibodies used for western blot (as shown in Table 2) were purchased commercially and used according to the manufacturer’s instructions.

**Table 2:** Antibodies used for Western blot

Antibody	Source	Cat Number
GAPDH	Proteintech	60004-1-Ig
IDH2	Proteintech	15932-1-AP
RNF185	Abcam	Ab181999
Flag-tag	AbHO	HOA012FL01

### Colony formation assay

MDA-MB-231 cells were seeded in six-well plates (1000 cells per well) and subjected to the indicated treatment for 2 weeks. Methanol was employed to fix the cells followed by crystal violet staining. The number of colonies was counted and analyzed.

### Measurement of relative NADPH levels

The NADPH production was measured using the NADP+/NADPH detection Kit (Beyotime Technology) according to the manufacture’s protocol.

### RNA-seq analysis

For RNA sequencing (RNA-Seq), total RNA of control and D-mannose treated cells was isolated using TRIZOL reagent. Each condition was prepared in triplicate for each RNAseq experiment. Library preparation using Illumina Miseq Reagent Kit and sequencing on an Illumina Hiseq2000 Truseq machine. Paired-end FASTQ sequences were aligned to the human genome using HISAT2 software. Differential gene expression analysis was performed with the DESeq2 package. The RNAseq data presented in the study were deposited in the GeneBank, https://www.ncbi.nlm.nih.gov/, accession number PRJNA918396.

### Statistics

Statistical analysis was performed using GraphPad Prism 8 software. Data in bar graphs represented as fold change or percentage relative to control with standard deviation of three independent experiments. Normally distributed data were analyzed using Student’s t-test. A p value less than 0.05 was considered statistically significant. Levels of significance were indicated as * p < 0.05, ** p < 0.01, *** p < 0.001, ****p < 0.0001.

## Results

### D-mannose downregulates IDH2 protein level

**Fig. 1:**
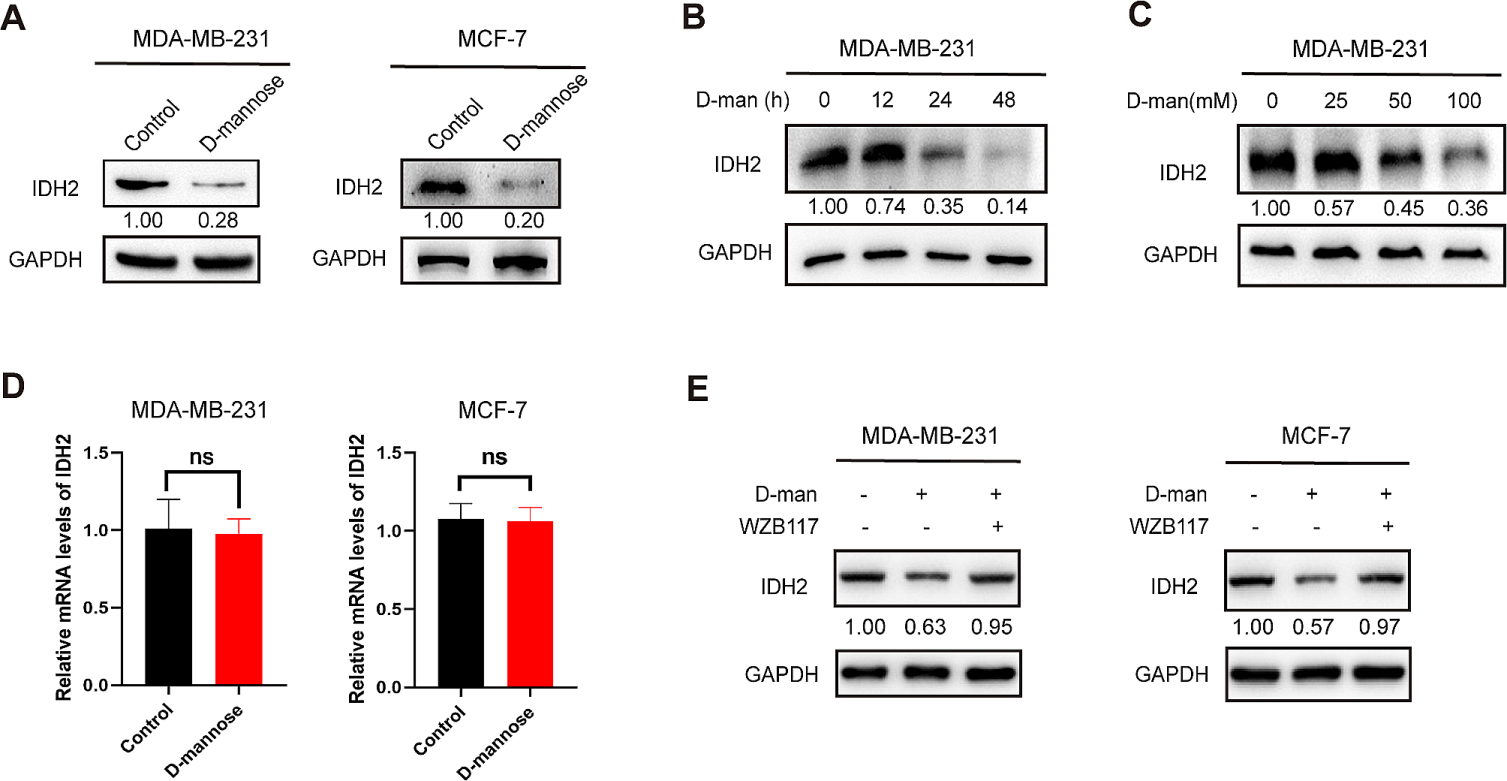
D-mannose decreased the protein level of IDH2. (A) Western blot analysis of IDH2 level in MDA-MB-231 cells and MCF-7 cells treated with or without D-mannose. (B) Western blot analysis of IDH2 level in MDA-MB-231 cells treated with 100 mM D-mannose for different time as indicated. (C) Western blot analysis of IDH2 level in MDA-MB-231 cells treated with different concentrations of D-mannose as indicated for 48 h. (D) qPCR analysis of IDH2 mRNA level in MDA-MB-231 cells and MCF-7 cells treated with or without D-mannose. Values are means ± SD from n = 3 independent experiments. Statistical differences were determined by t-test. ns, not significant. (E) Western blot analysis of IDH2 level in MDA-MB-231 cells and MCF-7 cells treated with or without D-mannose and WZB117.

It was reported that D-mannose treatment can increase reactive oxygen species (ROS) production [12], while IDH2 is important for the generation of NADPH, the crucial reducing factor. These findings prompted us to determine whether D-mannose can regulate IDH2. To test this hypothesis, we treated MDA-MB-231 cells with D-mannose and examined the expression level of IDH2. Interestingly, we found that D-mannose treatment significantly decreased the expression level of IDH2 in human breast cancer cell line MDA-MB-231 and MCF-7 (Fig. 1A). To confirm this result, MDA-MB-231 cells were treated with D-mannose for different time and dose. In line with previous results, D-mannose can reduce IDH2 in both time- and dose- dependent manners (Fig. 1, B and C). Notably, the mRNA level of IDH2 showed no response to D-mannose treatment in both cell lines (Fig. 1D). What’s more, block of D-mannose uptake into cells by GLUT transporter inhibitor WZB117 rescued the IDH2 degradation by D-mannose, which further validate our finding (Fig. 1E). In conclusion, D-mannose can downregulate IDH2 protein levels.

### D-mannose promotes the ubiquitination and degradation of IDH2

**Fig. 2:**
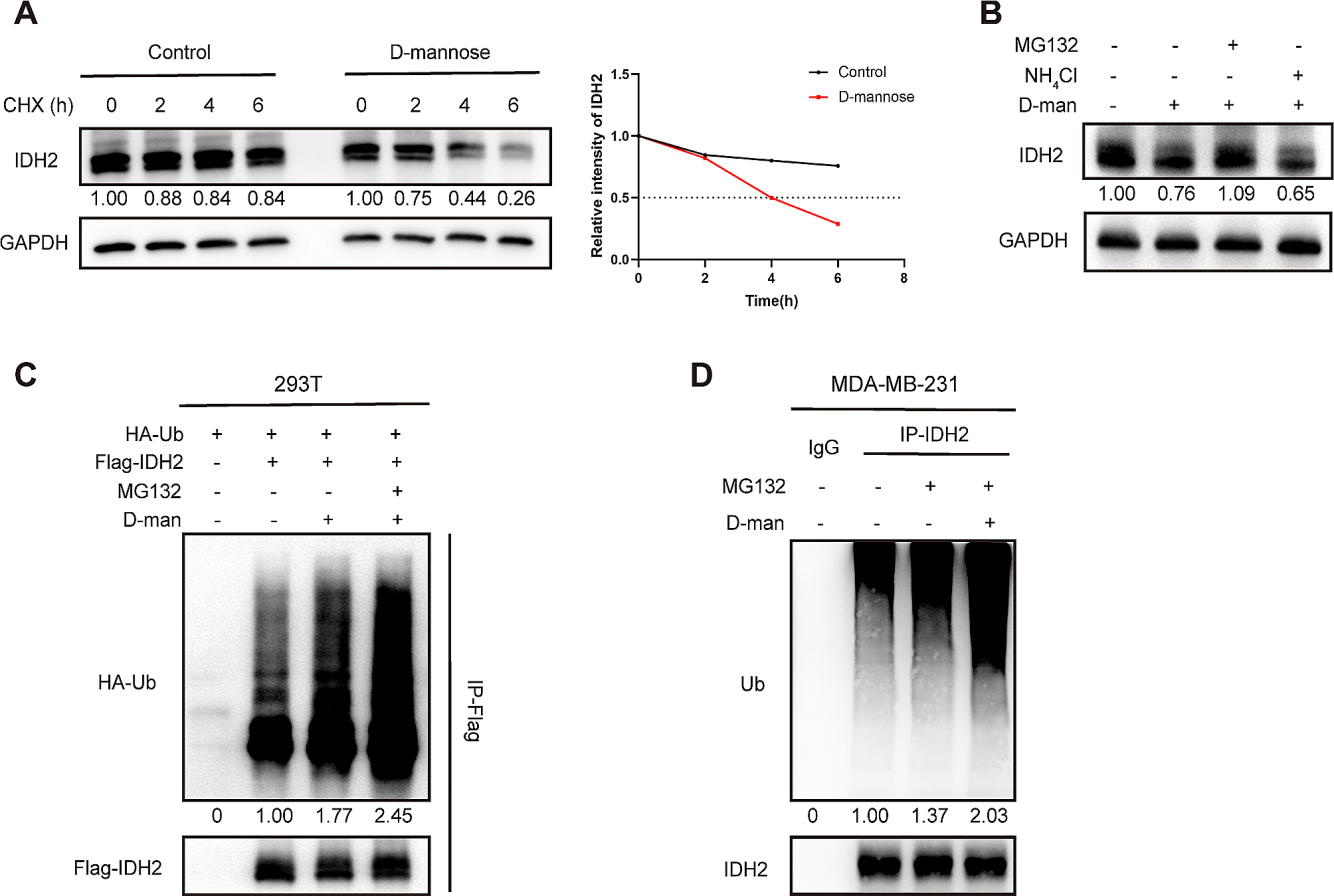
D-mannose promotes IDH2 ubiquitination and proteasomal degradation. (A) The half-life of IDH2 under D-mannose treatment was determined by CHX-chase assay in MDA-MB-231 cells. (B) Western blot analysis of IDH2 level in MDA-MB-231 cells treated with D-mannose in the absence or presence of proteasome inhibitor MG132, or lysosome inhibitor NH4Cl. (C) Western blot analysis of IDH2 ubiquitination level under D-mannose treatment in 293T cells with overexpressed Flag-IDH2. (D) Western blot analysis of IDH2 ubiquitination level under D-mannose treatment in MDA-MB-231 cells

Next, we determined whether D-mannose impairs the protein stability of IDH2. Cycloheximide (CHX) was used to switch off protein synthesis in MDA-MB-231 cells, followed by the detection of IDH2 protein levels in the subsequent 0, 2, 4, and 6 h. Results showed that D-mannose accelerated the degradation of IDH2 protein, and the half-life of IDH2 protein was much shorter under D-mannose treatment (Fig. 2A). To explore the pathway through which IDH2 protein level was decreased by D-mannose treatment, the D-mannose treated cells were subjected to proteasome inhibitor MG132 and lysosome inhibitor NH_4_Cl treatment, respectively, and the protein level of IDH2 was examined. As a result, we found that MG132 treatment blocked the D-mannose mediated IDH2 degradation (Fig. 2B). Furthermore, we evaluated the ubiquitination level of over-expressed Flag-IDH2 protein in 293T cells upon D-mannose treatment and found that D-mannose significantly increased the ubiquitination level of Flag-IDH2, especially under MG132 treatment (Fig. 2C). What’s more, to further illustrate our findings, ubiquitination level of endogenous IDH2 was detected, results showed that IDH2 ubiquitination level was upregulated under D-mannose treatment in MDA-MB-231 cells (Fig. 2D). Together, these results suggest that D-mannose mediates the degradation of IDH2 protein through the ubiquitin-proteasome pathway.

### D-mannose decreases the NADPH level and inhibits mammary cancer cell proliferation

**Fig. 3:**
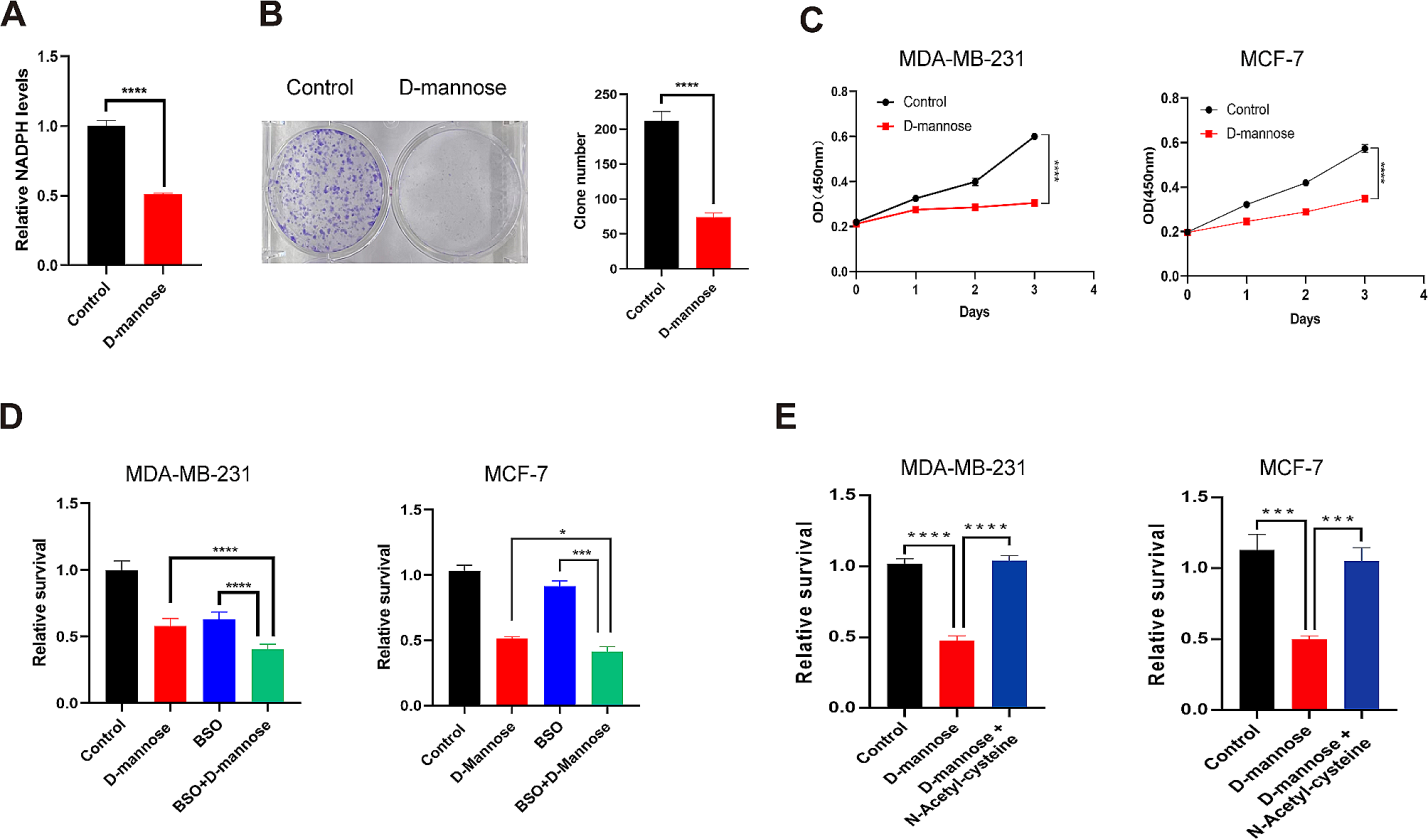
D-mannose synergizes with BSO to inhibit tumor cell proliferation. (A) Relative levels of NADPH in MDA-MB-231 cells treated with or without D-mannose. Values are means ± SD from n = 3 independent experiments. Statistical differences were determined by t-test. ****p < 0.0001. (B) Colony formation assay of the control and D-mannose treated MDA-MB-231 cells. Values are means ± SD from n = 3 independent experiments. Statistical differences were determined by t-test. ****p < 0.0001. (C) Cell proliferation assay of the control and D-mannose treated MDA-MB-231 cells and MCF-7 cells. Values are means ± SD from n = 3 independent experiments. Statistical differences were determined by t-test. ****p < 0.0001. (D) Relative survival of MDA-MB-231 cells and MCF-7 cells under the indicated treatments for 48 h. Values are means ± SD from n = 3 independent experiments. Statistical differences were determined by t-test. * p < 0.05, *** p < 0.001, ****p < 0.0001. (E) Relative survival of MDA-MB-231 cells and MCF-7 cells under the indicated treatments for 48 h. Values are means ± SD from n = 3 independent experiments. Statistical differences were determined by t-test. *** p < 0.001, ****p < 0.0001

While participating in the TCA cycle for energy production, IDH2 converts isocitrate to α-KG by reducing NADP + to NADPH. Therefore, we measured the levels of NADPH in MDA-MB-231 cells and found that D-mannose treatment can significantly decrease the cellular levels of NADPH (Fig. 3A). The colony formation and proliferation of cells were also tested, the results showed that D-mannose treatment significantly inhibited the colony formation of MDA-MB-231 cells (Fig. 3B). Consistently, the proliferation of both MDA-MB-231 cells and MCF-7 cells was also inhibited by D-mannose treatment (Fig. 3C). NADPH can serve as the donor of reductive power to neutralize the ROS accumulated in the rapid growth process in tumor cells. We thus evaluated the combination effect of a pro-oxidant, buthionine sulfoximine (BSO) and D-mannose, and found that, while both single agents decreased the tumor cell survival, the combination treatment showed the best effect of inhibition in both MDA-MB-231 cells and MCF-7 cells (Fig. 3D). To validate the role of ROS in D-mannose mediated suppression on cell proliferation, the anti-oxidant N-acetyl cysteine was supplemented into the D-mannose treated breast cancer cells and results showed that D-mannose mediated cell proliferation suppression was rescued by N-acetylcysteine treatment in both MDA-MB-231 and MCF-7 cells (Fig. 3E). Collectively, D-mannose can synergize with BSO to inhibit breast cancer cell proliferation through ROS accumulation.

### D-mannose promotes the expression of RNF185

**Fig. 4:**
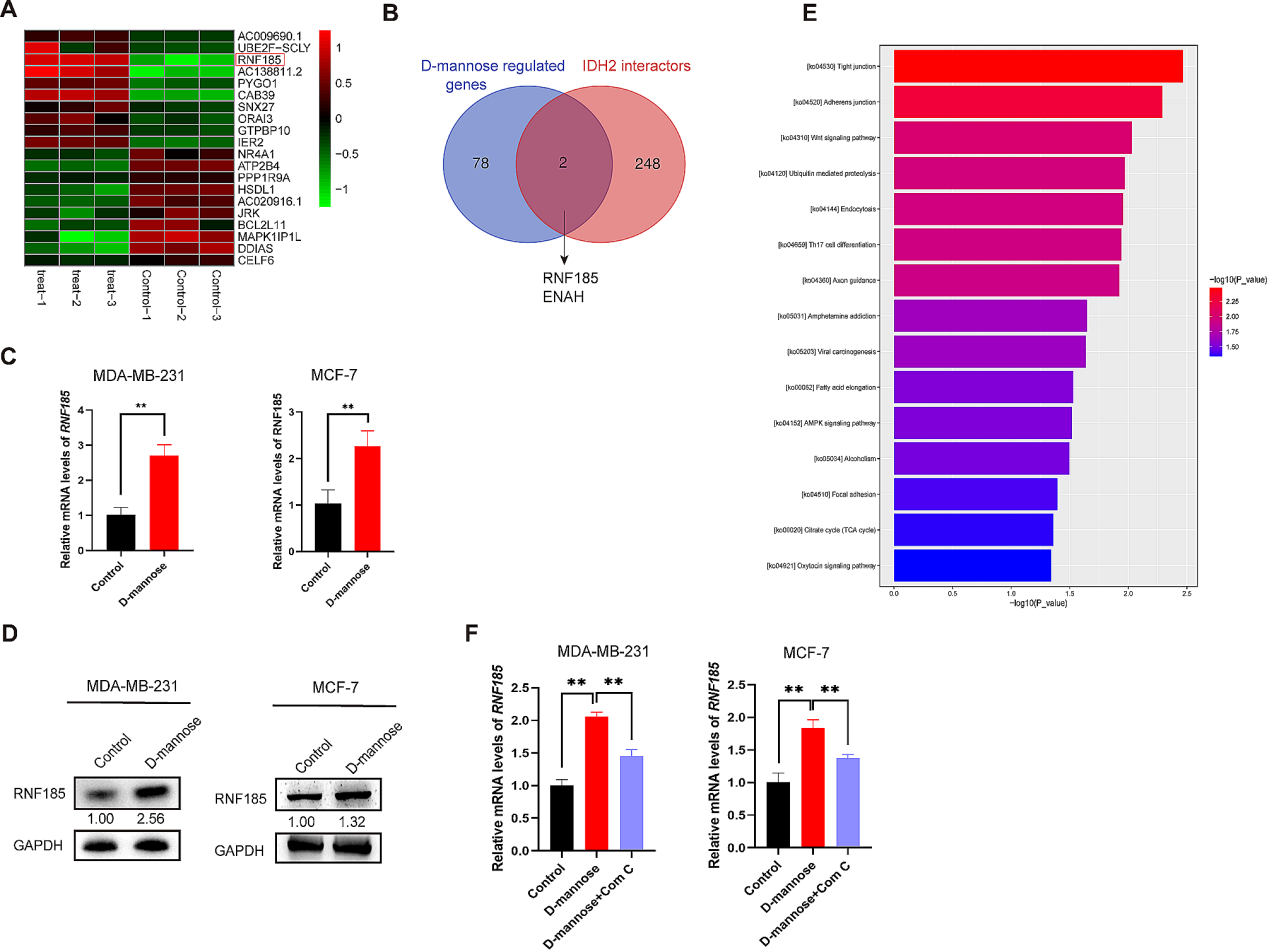
D-mannose elevates the expression of RNF185. (A) Heatmap showed the top 10 upregulated and top 10 downregulated genes by D-mannose treatment. (B) Venn diagram showed the cross analysis of RNA-seq data and BioGRID data. (C) qPCR analysis of RNF185 mRNA level in MDA-MB-231 cells and MCF-7 cells treated with or without D-mannose. Values are means ± SD from n = 3 independent experiments. Statistical differences were determined by t-test. ** p < 0.01. (D) Western blot analysis of RNF185 protein level in MDA-MB-231 cells and MCF-7 cells treated with or without D-mannose. (E) KEGG enrichment analysis of upregulated pathways in D-mannose treatment cells. (F) qPCR analysis of RNF185 mRNA level in MDA-MB-231 cells and MCF-7 cells treated with or without D-mannose and Compound C. Values are means ± SD from n = 3 independent experiments. Statistical differences were determined by t-test. ** p < 0.01

The E3 ubiquitin ligase plays an essential role in the protein ubiquitination and proteasomal degradation process by attaching ubiquitin to the lysine sites of targeted proteins [17]. Therefore, we tried to identify the E3 ubiquitin ligase involved in the D-mannose mediated IDH2 ubiquitination and degradation. The human mammary cancer cell line MDA-MB-231 was subjected to D-mannose treatment followed by RNA-seq analysis. The top 10 upregulated and top 10 downregulated genes by D-mannose treatment were identified and shown (Fig. 4A). Cross analysis of the RNA-seq identified D-mannose regulated genes and IDH2 interactome data from the BioGRID database highlighted the genes RNF185 and ENAH (Fig. 4B). Given that Ring Finger Protein 185 (RNF185) is an E3 ubiquitin ligase, which plays essential roles in the ubiquitination and proteasomal degradation of proteins, we focused on RNF185, which was sharply elevated after D-mannose treatment in MDA-MB-231 cells according to the RNA-seq data. (Fig. 4A). What’s more, the result of RNA-seq was validated in MDA-MB-231 cells and MCF-7 cells through qPCR, the mRNA level of RNF185 increased about 3 folds after D-mannose treatment (Fig. 4C). Consistently, the protein level of RNF185 also showed a significant increase (Fig. 4D). Then we tried to find the pathways involved in D-mannose mediated RNF185 upregulation, RNA-seq showed the upregulated pathways in D-mannose treatment cells (Fig. 4E). Among the upregulated pathways, AMPK signaling pathway caught our attention because it has been reported that D-mannose can activate AMPK in cancer cells [13, 16]. Therefore, we tested the effect of AMPK inhibitor on D-mannose mediated RNF185 upregulation and found that AMPK inhibitor compound C can inhibit the upregulation of RNF185 by D-mannose (Fig. 4F), suggesting an important role of AMPK signaling in this process. Taken together, D-mannose increases the expression of RNF185 in breast cancer cells through AMPK acitivation.

### D-mannose promotes the degradation of IDH2 through RNF185

**Fig. 5:**
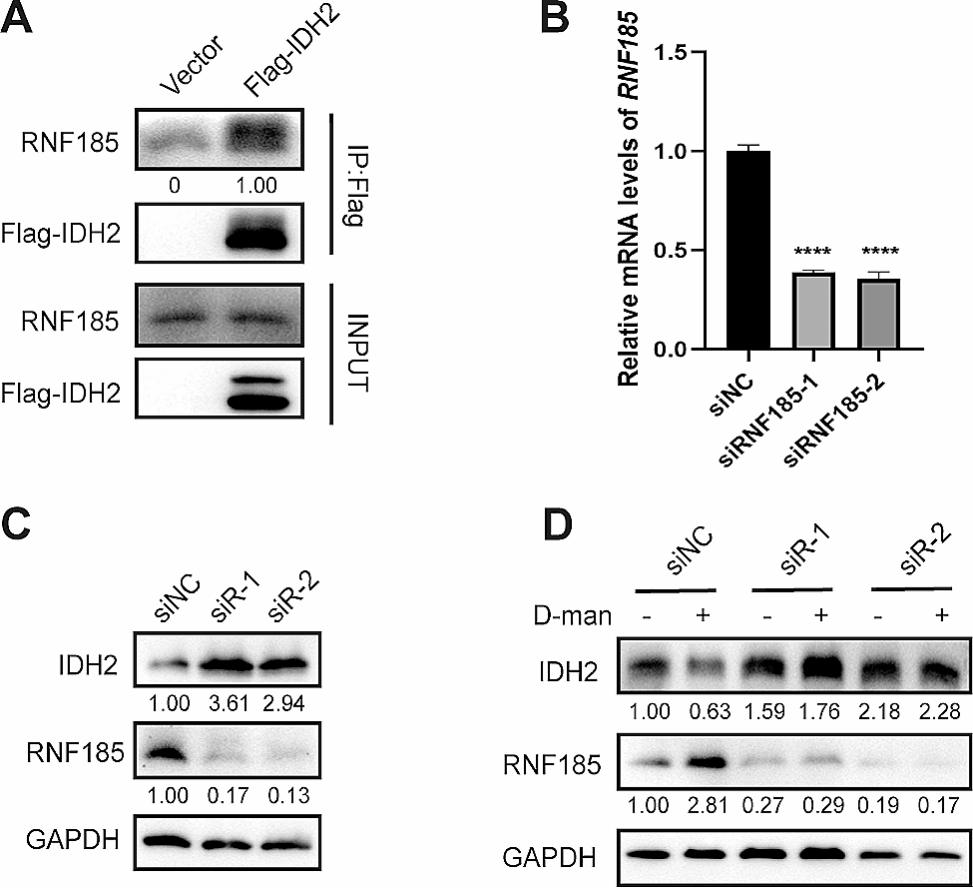
RNF185 binds with and degrades IDH2 under D-mannose treatment. (A) The interaction between Flag-IDH2 and RNF185 in 293T cells was determined by co-IP and western blot. (B) qPCR analysis of RNF185 mRNA level in MDA-MB-231 cells transfected with control or siRNF185. Values are means ± SD from n = 3 independent experiments. Statistical differences were determined by t-test. ****p < 0.0001. (C)Western blot analysis of RNF185 and IDH2 protein levels in MDA-MB-231 cells transfected with control or siRNF185. (D)Western blot analysis of RNF185 and IDH2 protein levels in MDA-MB-231 cells transfected with control or siRNF185 and treated with or without D-mannose

We then wondered whether the E3 ubiquitin ligase RNF185 plays a role in the D-mannose mediated IDH2 ubiquitination and degradation. The co-immunoprecipitation was conducted in 293T cells to test if there was any interaction between RNF185 and Flag-tagged IDH2. The result showed that RNF185 can bind to Flag-IDH2 (Fig. 5A). Furthermore, small interfering RNAs (siRNAs) were developed to disturb the expression of RNF185(Fig. 5B) and the effect of RNF185 knockdown on IDH2 protein level was examined. The result showed that knockdown of RNF185 led to a significant increase of IDH2 protein level (Fig. 5C). What’s more, knockdown of RNF185 effectively blocked D-mannose mediated IDH2 degradation (Fig. 5D). Taken together, these results suggest that RNF185 can directly bind to IDH2 and promote its degradation under D-mannose treatment.

### RNF185 plays a key role in the D-mannose mediated inhibition of tumor cell proliferation through IDH2 degradation

**Fig. 6:**
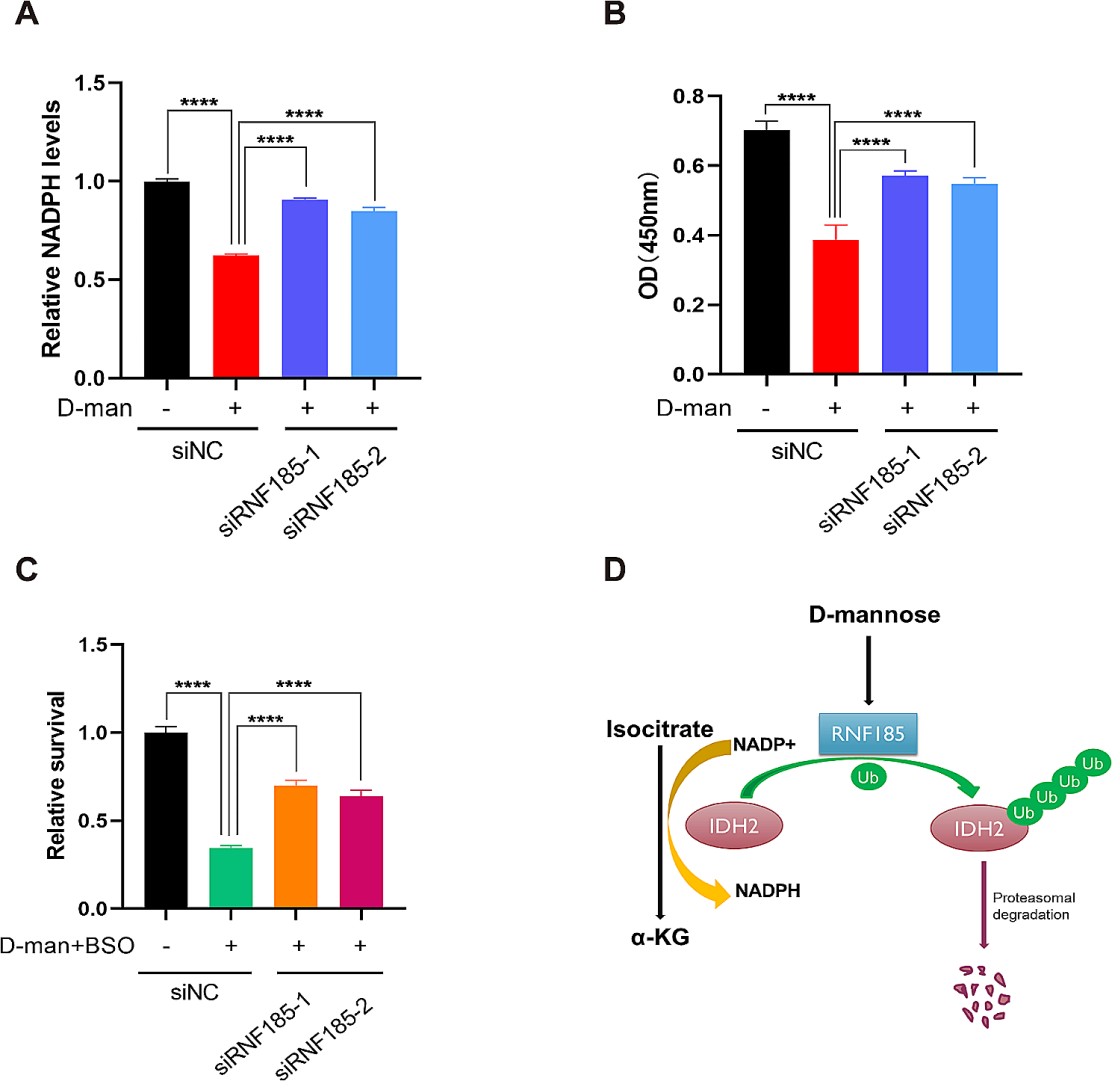
Knockdown of RNF185 abrogates the effect of D-mannose on tumor cells. (A) Relative levels of NADPH in MDA-MB-231 cells with indicated treatment. Values are means ± SD from n = 3 independent experiments. Statistical differences were determined by t-test. ****p < 0.0001. (B) Cell proliferation analysis of MDA-MB-231 cells with indicated treatment. Values are means ± SD from n = 3 independent experiments. Statistical differences were determined by t-test. ****p < 0.0001. (C) Relative survival analysis of MDA-MB-231 cells under the indicated treatments for 48 h. Values are means ± SD from n = 3 independent experiments. Statistical differences were determined by t-test. ****p < 0.0001. (D) Schematic representation of the mechanism under D-mannose mediated IDH2 degradation

To further validate the role of RNF185-IDH2 axis in D-mannose mediated inhibition of tumor cell proliferation, we knocked down RNF185 in MDA-MB-231 cells and examined its effects on NADPH production and cell proliferation. The results showed that D-mannose mediated decrease of cellular NADPH level can be recovered by knockdown of RNF185(Fig. 6A). Similarly, the inhibition of tumor cell proliferation by D-mannose was rescued under RNF185 knockdown (Fig. 6B). Furthermore, when RNF185 was knocked down, the inhibition effect of BSO and D-mannose combination on tumor cells was partially abrogated, and the relative survival of tumor cells showed significant improvement (Fig. 6C). Together, we concluded that D-mannose mediated RNF185 upregulation plays a key role in IDH2 degradation and tumor cell inhibition (Fig. 6D).

## Discussion

Wild-type IDH2 has been identified as a prognosis marker and potential therapeutic target in breast cancer. Here we demonstrated that, in MDA-MB-231 cells, wild-type IDH2 protein can be degraded through the ubiquitination-proteasome pathway, and D-mannose can promote this process through upregulation of the E3 ligase RNF185 (Fig. 6D). While promoting the degradation of IDH2, D-mannose can inhibit the production of NADPH as well, thus rendering the tumor cells more sensitive to the pro-oxidant BSO treatment. In our study, a novel axis for the regulation of IDH2 protein was illustrated, which may contribute to the exploration of IDH2 targeted therapies, and D-mannose may serve as a potential drug targeting IDH2 protein. What’s more, the effect of D-mannose on the mutant IDH2 also warrants evaluation.

Through the RNA-seq analysis, we found that RNF185 can be positively regulated by D-mannose treatment. Given that RNF185, as an E3 ligase, has the potential to regulate more proteins except for IDH2, we speculate that D-mannose may also act on other targets of RNF185, which deserves further investigation. In this study, we have revealed that D-mannose inhibits the proliferation of breast cancer cells via RNF185-IDH2 axis, however, the multiplex pathways involved in D-mannose treatment haven’t been thoroughly deciphered yet, especially the mechanism underlying D-mannose induced RNF185 upregulation need to be further explored.

Previous studies have illustrated different roles of D-mannose in cancer. Here, we not only validated the effect of D-mannose on the inhibition of breast cancer, but also revealed a novel mechanism of this effect. A previously unknown target of D-mannose, IDH2, was identified, which broadens our knowledge about the effect of D-mannose in the context of tumor, especially tumor metabolism. Our study demonstrated that D-mannose not only directly participates in the process of cellular metabolism as a metabolite, but also functions in regulating the stability of a metabolic enzyme important for tumor growth.

## Conclusions

In summary, our study revealed a novel post-transcriptional regulation of IDH2 protein by the E3 ligase RNF185, and illustrated the function of D-mannose on regulating RNF185-IDH2 axis to inhibit the proliferation of breast cancer cells. Our findings further address the possibility of D-mannose as a drug for cancer treatment, and may contribute to the exploration of IDH2 targeted therapies.

## Data Availability

The data that supports the findings of this study are available in this article. The RNAseq data presented in the study are deposited in the GeneBank (https://www.ncbi.nlm.nih.gov/), accession number PRJNA918396.

## Abbreviations

IDH2: Isocitrate dehydrogenase 2
RNF185: Ring finger protein 185
ROS: Reactive oxygen species
α-KG: α-ketoglutarate
2-HG: 2-hydroxyglutarate
